# Long-term studies reveal major environmental factors driving zooplankton dynamics and periodicities in the Black Sea coastal zooplankton

**DOI:** 10.7717/peerj.7588

**Published:** 2019-09-27

**Authors:** Alexander L. Vereshchaka, Liudmila L. Anokhina, Taisiya A. Lukasheva, Anastasiia A. Lunina

**Affiliations:** Shirshov Institute of Oceanology, Russian Academy of Sciences, Moscow, Russia

**Keywords:** Black sea, Environmental factor, Coastal ecosystems, Zooplankton, Periodicity

## Abstract

**Background:**

The development and management of shelf-sea ecosystems require a holistic understanding of the factors that influence the zooplankton structure and ecosystem functions. The Black Sea is an example of such areas influenced by eutrophication, overfishing, climate variability, invasions of the ctenophores *Mnemiopsis leidyi* followed by *Beroe ovata*. Thus, there is a set of principal factors which may influence and explain periodicities in the Black Sea ecosystem.

**Methods:**

We analysed a total of 918 samples taken from 1991 to 2017 with intervals of 10 days. Taxa were identified to species, their abundance and biomass were calculated. We tested 12 environmental factors, which may explain zooplankton distribution: temperature, productivity-linked factors (surface chlorophyll as a proxi), wind, turbidity, lowest winter temperature, and concentration of the ctenophore *M. leidyi*. We used canonical correspondence analyses to find the dominant environmental factors and further regression analyses to retrieve dependences of plankton biomass on the major factors. Periodicities were assessed with the use of the Continuous wavelet transform and tested with use of One-way ANOSIM and PERMANOVA. The distances between ecosystem states in different years were assessed using non-metric multidimensional scaling.

**Results:**

Currently, temperature and productivity are the major environmental factors driving zooplankton dynamics. Not long ago, before 1999, abundance of *M. leidyi* was one of the major factors explaining the zooplankton variance. Spectral analysis of species abundances revealed a 4-year transitional period in 1999–2002 (not reported before) when ecosystem adapted to a new invader *B. ovata*. Statistically robust 2- and 3-year periodicities were retrieved for most plankton taxa and some benthic larvae. We found robust correlations between temperature and surface chlorophyll concentration on one side and plankton abundances and biomass on the other, and retrieved multivariate regressions, which may have a prognostic value.

## Introduction

Zooplankton on continental shelves represents a significant intermediary in the transfer of energy and matter from phytoplankton to the wider ecosystem. The development and management of shelf-sea ecosystems require a holistic understanding of the factors that influence the zooplankton community structure and the ecosystem functions. The Black Sea is an example of such areas influenced by eutrophication, overfishing, climate variability, and biological invasions. The sea is a semi-enclosed meromictic temperate basin with average depth 1,253 m, 2,212 m maximal depth, and narrow shelf that in the Southern and Eastern parts rarely exceeds 20 km; water circulation is dominated by a boundary current with cross-shelf water exchange and lateral mixing (Özsoy & Ünlüata, 1997; Sur, Ozsoy & Unluata, 1994). The Black Sea is characterised by a rather high productivity causing high edible zooplankton stocks but relatively low biodiversity (Mordukhai-Boltovskoi, 1972). Since the second half of the 20th century, the plankton of the Black Sea has been under biotic and abiotic pressures such as eutrophication (Petranu, 1997; Zaitsev & Alexandrov, 1998; Humborg et al., 1997; Yunev, Moncheva & Carstensen, 2005), overfishing (Daskalov, 2002; Daskalov et al., 2007; Llope et al., 2011), climate variability (Oguz et al., 2003; Oguz, Dippner & Kaymaz, 2006), invasions of the ctenophores *Mnemiopsis leidyi* followed by *Beroe ovata* (Vinogradov et al., 1991; Shiganova et al., 2018). *M. leidyi* appeared in the late 1980s and caused a degradation of the pelagic ecosystem; further invasion of *B. ovata* (late 1990s) feeding on *M. leidyi* gave the ecosystems a chance to recover (Finenko et al., 2003; Shiganova et al., 2004; Arashkevich et al., 2008; Vinogradov et al., 2005). These processes were documented by the open water surveys cited above but only in basic aspects: deeper insight requires more regular and long-term observations with higher temporal resolution.

**Figure 1:**
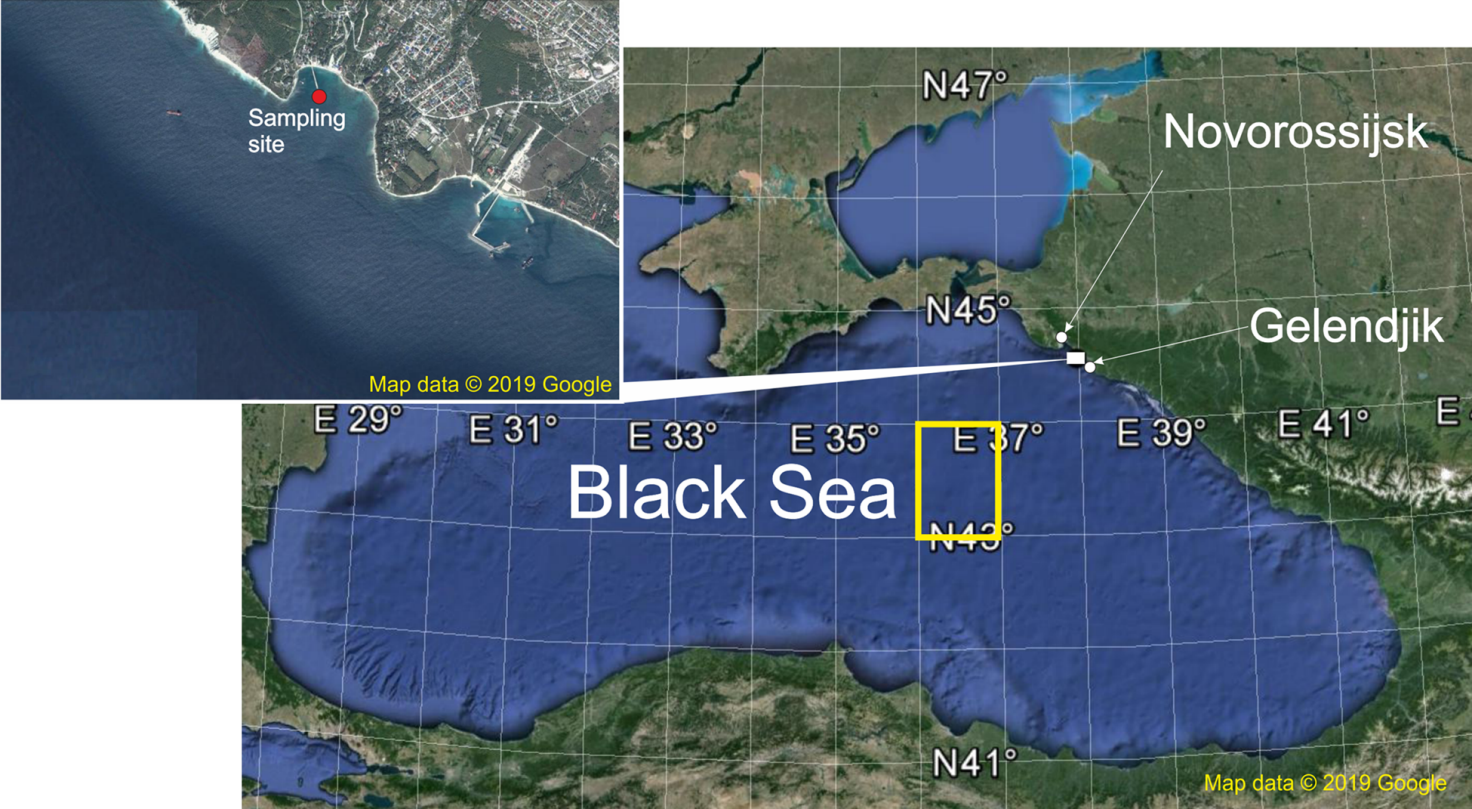
The sampling area. Red point shows sampling site, yellow rectangle indicates areas of the surface chlorophyll averaging. Map data © 2019 Google.

We made year-round long-term observations with a time gap of 10 days for 27 years, from 1991 to 2017, near the Golubaya Bay (Fig. 1). We started with analysis of the benthopelagic and benthoneustonic fauna, have yielded regularities in diurnal dynamics of these plankton groups, and assessed environmental factors driving this dynamics (Vereshchaka & Anokhina, 2014, 2015, 2017a, 2017b). Here, we make the next step and analyse temporal variability of all plankton taxa with the focus on the holoplanktonic fauna.

The first goal of the paper is to assess major environmental factors driving zooplankton dynamics during the period of observations. Among them we consider:

- Temperature. As in other temperate areas, the Black Sea ecosystems are exposed to seasonal environmental changes, which result in regular changes in faunistic composition and biomass.
- Surface chlorophyll‑a concentration (*Chl*). This factor is used as a proxy for primary production, which provides the energy basement for plankton communities and may explain a significant part of plankton biomass variance on a large scale, even in the deep ocean (Vereshchaka et al., 2016, 2017; Vereshchaka, Lunina & Sutton, 2019).
- The lowest winter temperature. This parameter was hypothesised to be a driver of the strength of the spring phytoplankton bloom (Mikaelyan et al., 2017).
- Strong northeast wind (Bora). This local factor causes negative surge and a decrease in the surface water temperature of 1–2 °C, and thus may affect the composition of the coastal plankton communities.
- Turbidity. This factor is locally linked to longstanding south winds resulting in storms. Increased turbidity is known to affect composition and biomass of the pelagic and benthopelagic communities in marine habitats (Vereshchaka, 1995).
- Concentration of *M. leidyi*. This factors may be considered as a significant environmental owing to the impact on the mesoplankton.

Environmental factors above cause changes in coastal communities at various temporal scales and the second goal of the paper is to retrieve periodicities and fluctuations of the main plankton components. We will analyse periodicities at several scales: long-term periodicities (several years or more), short-term inter-annual periodicities (few years), and within-year periodicities.

Having found strong environmental signals driving composition of coastal mesoplankton, we will further make multivariate regression analyses in order to find possible correlations between these factors on one side and abundances of individual taxa and total zooplankton biomass on the other. Retrieval of such regressions is our third goal: the regressions, if robust, may have a prognostic value and can be used for future forecasts.

## Methods

### Study site, sample collection and analysis

Samples were taken at the Northeast coast of the Black Sea in the Golubaja (‘Blue’) Bay near Novorossijsk (Fig. 1) from 4 June 1991 to 14 December 2017 with intervals of 10 days. This is an open bay characterised by typical environmental parameters for the northeast coast of the Black Sea (Lebedeva et al., 2003; Vinogradov et al., 2005; Vereshchaka & Anokhina, 2014, 2015, 2017a, 2017b). Under stormy conditions, which occurred mainly in the winter period, sampling was delayed 1–3 days. A total of 918 samples were thus collected, 32–37 per year except the starting 1991 when only 20 samples were taken. During the period of observations there were two remarkable events: satellite chlorophyll data (*Chl*) became available since 1998 and the ctenophore *B. ovata* appeared in 1999.

We sampled the zero to seven m layer at a distance of 170 m from the coast with a Judey net (mouth area 0.1 m^2^, mesh size 180 µm), towed at 50 cm s^−1^. All samples were taken at 10:00 am. Ctenophores and medusas, including ova and youngest larvae, were separated from other organisms and measured instantly (*M. leidyi* with lobes). Samples were preserved in 4% seawater/formaldehyde solution and identified to species level using a stereomicroscope. Some taxa such as Harpacticoida, Rotifera, larvae of Decapoda, Cirripedia and Polychaeta were not always identified to species level and were grouped to higher taxa. A few species, which define the ‘face’ of the recent Black Sea plankton ecosystem, were recorded in more detail. *M. leidyi* was divided into three size groups: <2, 2–10, and >10 mm, which we conventionally call larvae, juveniles and adults, respectively; ova were excluded from the analyses, as they were often destroyed and could not be recorded representatively. *B. ovata* was identified to the developmental stage (ovum, larva, adult), as was *Aurelia aurita* (ephyra, adult). Species were identified with the use of Mordukhai-Boltovskoi (1968, 1969, 1972); recent taxonomy was checked with the Word Register of Marine Species (http://www.marinespecies.org). Taxa were divided in two principal groups: mero- and holoplanktonic; the list of dominant taxa is presented in Table 1.

**Table 1:** Dominant taxa included in CCA analyses (alphabetical order for holo- and meroplankton separately), abbreviations, and their representation in samples.

Taxa	Abbreviations in CCAs (Figs. 2–3)	Average abundance (ind m^−3^) in samples	Average biomass (mg m^−3^) in samples
HOLOPLANKTON			
*Acartia clausi*	*A. clausi*	97.57	0.0140
*Aurelia aurita*, adults	*A. aurita*	0.05	0.0858
*Aurelia aurita*, larvae	L. Aurelia	0.04	0.0004
*Beroe ovata*, adults	*B. ovata*	0.06	0.1359
*Beroe ovata*, larvae	L. *B. ovata*	2.04	0.0002
*Beroe ovata*, ova	Ova *B. ovata*	0.28	0.0000
*Calanus euxinus*	*C. euxinus*	0.56	0.0012
*Centropages ponticus*	*C. ponticus*	27.23	0.0021
Copepoda, nauplii	*N. Copepoda*	30.20	0.0002
Copepoda, ova	Ova copepoda	2.80	0.0001
*Evadne nordmanni*	Mnem >2	1.66	0.0002
Hydrozoa	Hydrozoa	3.51	0.0001
*Mnemiopsis leidyi* < 2 mm	L. Mnemiopsis	4.93	0.2342
*Mnemiopsis leidyi* 2–10 mm	Mnemiopsis 2–10 mm	1.14	0.2432
*Mnemiopsis leidyi* > 10 mm	Mnemiopsis >10 mm	0.53	0.3076
*Noctiluca scintillans*	*N. scintillans*	556.56	0.1645
*Oikopleura dioica*	*O. dioica*	22.20	0.0010
*Oithona davisae*	*O. davisae*	3.18	0.0002
*Oithona similis*	*O. similis*	13.90	0.0009
*Paracalanus parvus*	*P. parvus*	53.52	0.0147
*Parasagitta elegans*	*P. elegans*	10.79	0.0003
*Penilia avirostris*	*P. avirostris*	149.74	0.0208
*Pleopis polyphemoides*	*P. polyphemoides*	62.04	0.0007
*Pseudevadne tergestina*	*P. tergestina*	16.02	0.0008
Rotifera	Rotifera	25.71	0.0009
Total holoplankton	BH	1,112.25	1.2300
MEROPLANKTON			
Amphipoda	Amphipoda	0.12	0.0003
Bivalvia, larvae	L. Bivalvia	57.17	0.0059
Cirripedia, cypris stage	L. Cirripedia	2.96	0.0005
Cirripedia, nauplii	N. Cirripedia	40.54	0.0062
Decapoda, larvae	Decapoda	1.77	0.0045
Gastropoda, larvae	L. Gastropoda	24.71	0.0040
Harpacticoida	Harpacticoida	4.39	0.0007
Polychaeta, larvae	L. Polychaeta	23.19	0.0173
Pisces, larvae	L. Pisces	0.09	0.0003
Pisces, ova	Ova pisces	0.46	0.0003
Total meroplankton	BB	176.25	0.0400
Total plankton	BT	1,288.50	1.2700

We always filtered 0.7 m^−3^, which was satisfactory for abundant mesoplankton but ostensibly not enough for the larger size groups (ctenophores >10 mm and adult *A. aurita*). However, in the periods of bloom these species were regularly present in the catches. In order not to loose the data we cautiously included these groups in the analyses.

For each taxon, the numbers of specimens in the sample and individual sizes (length) were recorded with a precision of 0.1 mm. On the basis of this primary dataset the individual weights, species abundance and biomass, and the total abundance and wet biomass were calculated with the use of the plankton sample treatment programme PLANKTY (Dyakonov, 2002). When abundances and biomass (individuals and wet weight per m^3^) were calculated, the filtration coefficient was assumed to be 1.0.

### Environmentals scoring

Temperature was used as a proxy for the biological seasonality and measured immediately after sampling with a precision of 0.1 °C.

Surface chlorophyll‑a concentration (*Chl*) data were taken from Aqua MODIS (level 3, four-km resolution) from 2003 to 2015. Before this period, from 1997 to 2002, *Chl* data derived from SeaWiFS (level 3, nine km resolution). Here, we tested two temporal and two spatial scales of the averaging. We averaged *Chl* over 1 year and over 1 month preceding sampling.

The spatial scales merit remarks. The coastal waters along the Caucasian shelf are strongly affected by riverine loads of nutrients and organic matter, which mask the variability inherent for the whole basin (Zavialov et al., 2014) and the proper *Chl* concentration. The sea regions with the most representative characteristics of the open waters ecosystems are the western and eastern central areas, where the most intensive bottom-up flow of nutrients to the photic zone defines the ecosystem in the most ‘pure’ form (Oğuz, 2008). Therefore, on the spatial scale, we averaged *Chl* over two rectangular areas: 44.33–44.56°N and 37.75–38.00°E (642 km^2^ around Gelenjik) and 43–44°N and 36–37.00°E (9,717 km^2^, open waters of the eastern Black Sea) (Fig. 1).

We thus tested four possible combinations: (1) *Chl* averaged over the smaller area over 1 month, (2) *Chl* averaged over the smaller area over 1 year, (3) *Chl* averaged over the larger area over 1 month, and (4) *Chl* averaged over the larger area over 1 year.

The lowest winter temperature was obtained from Aqua MODIS satellite monthly images from 2003 to 2017 (http://oceancolor.gsfc.nasa.gov/cgi/l3). From 1991 to 2002 the monthly values were taken from NCEP reanalysis (http://www.esrl.noaa.gov/psd/data/timeseries).

Wind (Bora) was treated as a binary parameter: either absent or present in a period of 0–24 h before sampling.

Turbidity was also treated as binary parameter and assessed visually during the sampling.

Concentration of *M. leidyi*. We used three various values: abundances of larvae, juveniles, and adults.

### Canonical correspondence analyses

We performed canonical correspondence analyse (CCAs) (Ter Braak, 1986; Legendre & Legendre, 1998) of a taxa matrix, where each taxon had values for several environmental variables (Legendre & Legendre, 1998). The ordination axes were linear combinations of the environmental variables, thus representing direct gradients, and taxa abundances were considered to be a response to these gradients. The implementation followed the eigen analysis algorithm (Legendre & Legendre, 1998); the ordinations were given as site scores. Environmental variables were plotted as correlations with site scores.

The satellite *Chl* data were available since the second half of 1997 (1-year averaging was therefore possible since the middle of 1998 only), while the *M. leidyi* signal weakened since 1999, when the predator *B. ovata* appeared in the eastern part of the Black Sea. Therefore, direct comparison of signals linked to productivity and to *M. leidyi* concentration when it was maximal was not possible. Therefore, we made four different analyses.

In Analysis 1 we included all 12 environmental factors and analysed the dataset for the period 1998–2017 in order to find the strongest signals. We understood that *M. leidyi* signal in this period was weaker that before.

Analysis 2 we used same data set as in Analysis 1 but transferred *M. leidyi*-linked parameters from environmental factors to a set of the dependent variables to see how the whole plankton community including *M. leidyi* responded to the abiotic factors and to *Chl*.

In Analysis 3 we removed the strongest signal(s) retrieved in Analysis 2 in order to see the contribution of minor signals at a better resolution.

In Analysis 4 we analysed the dataset 1991–1998 without *Chl* data, as the satellites SeaWiFS and Aqua MODIS did not act at that time. The *M. leidyi* signal was probably strongest because the population grew without supressing effect of *B. ovata*.

Thus, we had two analyses (1 and 4) with *M. leidyi* considered as additional environmental factors and incomplete set of taxa (*M. leidyi* excluded) plus two more analyses (2 and 3) with typical environmental factors (abiotic and *Chl*) and the whole set of the plankton taxa as dependent variables.

### Statistical procedures

We used a continuous wavelet transform (CWT) for an assessment of periodicities. This analytical method inspect a dataset at small, intermediate and large scales simultaneously (Prokoph, Fowler & Patterson, 2000). The shape of the mother wavelet was set to Morlet (wavenumber 6). The significance level in CWT corresponded to *p* = 0.05. The ‘Lag’ value was set to 0 to specify a white-noise model.

Hypotheses of long-term periodicities in the Black sea ecosystem were tested with the use of One-way ANOSIM and PERMANOVA, the significance was computed by permutation of group membership set to 9,999 replicates. The distances between ecosystem states in different years were assessed using non-metric multidimensional scaling (MDS), which was based on a distance matrix computed with Bray–Curtis similarity index for abundance data (Taguchi & Oono, 2005).

We used the linear model for the bivariate regressions and Ordinary Least Squares algorithm, the permutation test on correlation (*r*^2^) used 9,999 replicates. The coefficients (intercept, and slope for each independent variate) were presented with their estimated standard errors and *t*-tests.

Calculations and analyses were carried out with the use of Excel, STATISTICA and PAST 3.04 (Hammer, Harper & Ryan, 2001). Correlations were considered significant if *p* < 0.05.

## Results

### Taxa abundance

**Figure 2:**
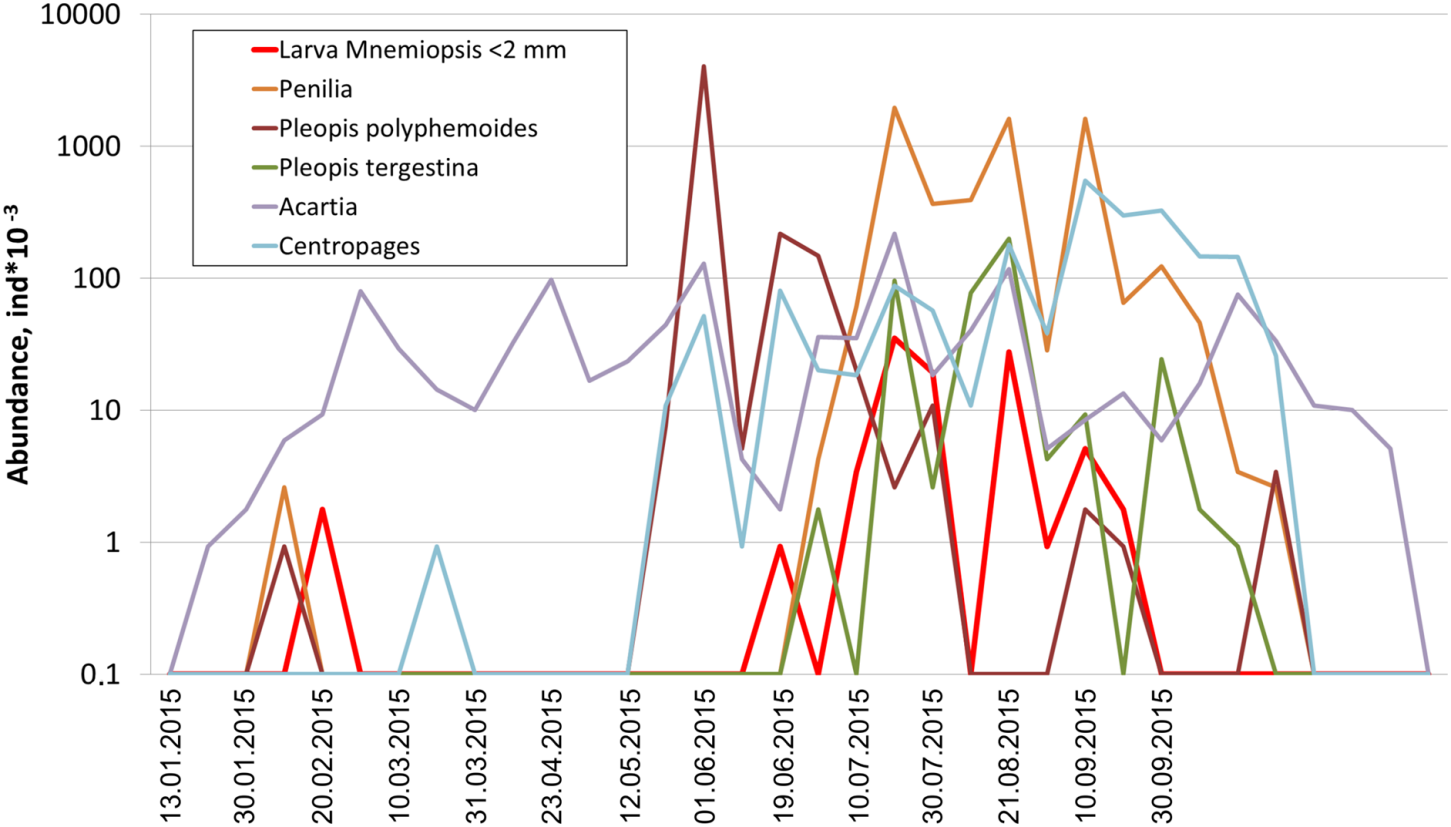
Seasonal dynamics of dominant taxa during the year 2015, vertical axis logarithmic.

Among holoplanktonic species, the most abundant were *Noctiluca scintillans*, followed by the copepods *(Acartia clausi, Centropages ponticus, Paracalanus parvus* and unidentified nauplii), the branchiopods (*Pleopis polyphemoides* and *Pseudevadne tergestina)*, the larvacean *Oikopleura dioica*, ctenophores, medusa, and Rotifera (Table 1). Meroplankton was dominated by the larvae of bivalves, gastropods, polychaetes and cirripeds (Table 1). In terms of biomass, total plankton was dominated by the holoplankton (1.23 mg m^–3^ on average, that is, 97% of the cotal biomass), while contribution of the meroplankton was negligible (0.04 mg m^–3^ on average, that is, 3%). An example of actual annual dynamics of dominant taxa is shown in Fig. 2.

### Multivariative CCAs

**Figure 3:**
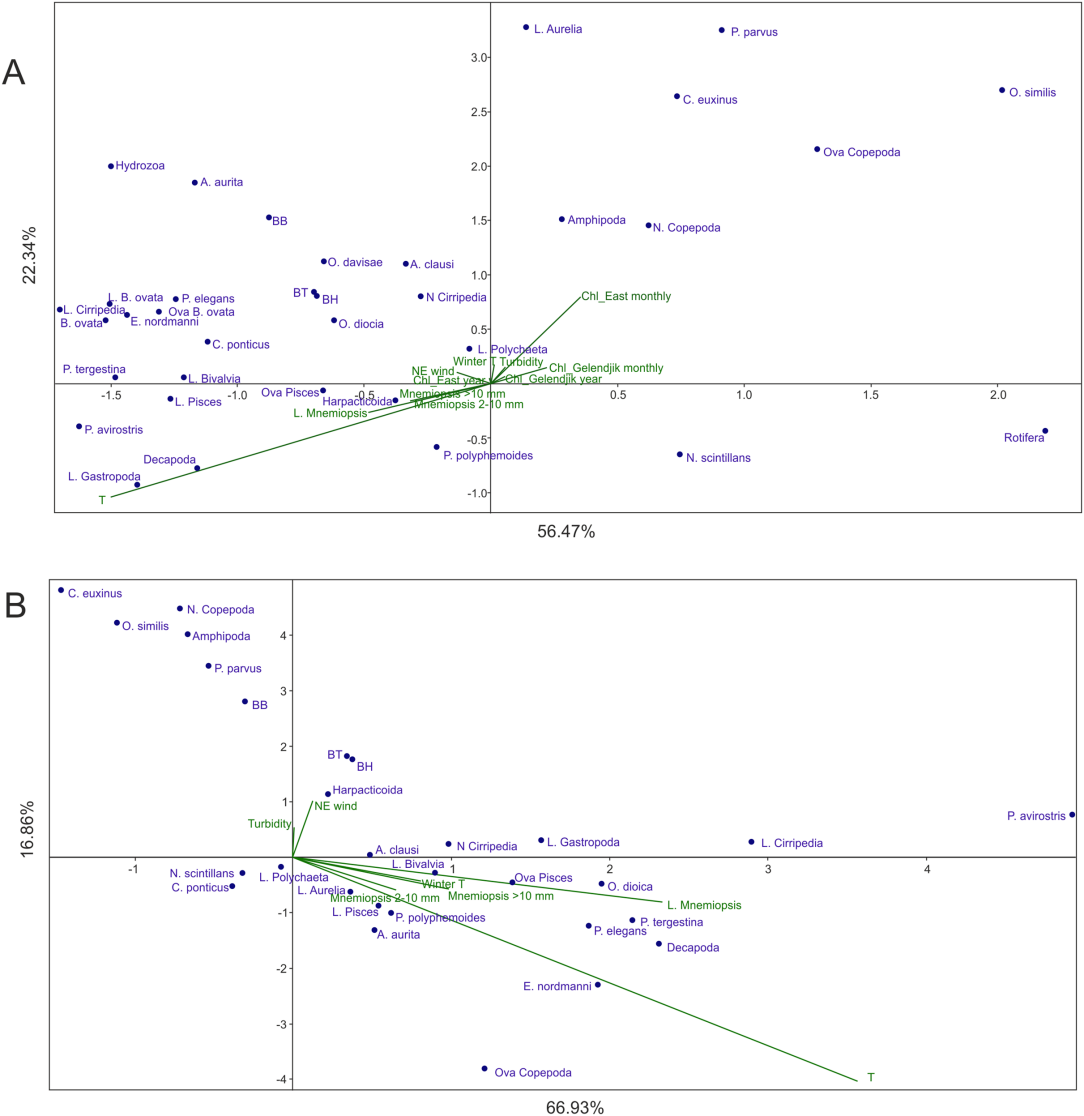
CCAs with abundances of *M. leidyi* considered as environmental factors. (A) Samples of 1998–2017 with all 12 environmental variables included, (B) samples of 1991–1998, the period of the uncontrolled *M. leidyi* bloom, the satellites SeaWiFS and Aqua MODIS did not act. Two first axes (F) with respective explained variance represented. Taxa code see in Table 2; BH, BB, and BT: biomass of the holoplankton, meroplankton, and total plankton biomass, respectively. Environmental variables: temperature (T), *Chl* averaged over the Gelendjik area over 1 month (Chl Gelendjik monthly), (2) *Chl* averaged over the Gelendjik area over 1 year (Chl Gelendjik year), *Chl* averaged over the central East area over 1 month (Chl East monthly), *Chl* averaged over the central East area over 1 year (Chl East year), Northeast wind (NE wind), turbidity (Turbid), lowest winter temperature (Winter T), abundances of larvae (L. Mnemiopsis), juveniles (Mnemiopsis 2–10 mm), and adults (Mnemiopsis >10 mm) of *M. leidyi*.

In Analysis 1 (all factors included), the first factor (*x*-axis) was mainly linked to temperature, while the second factor (*y*-axis) was primarily associated with *Chl* averaged over 1 month and over the open East Black Sea (Fig. 3A). *M. leidyi*-linked factors were nearly coaxial to temperature; the larvae of *M. leidyi* provided the strongest signal but even this signal was nearly threefold weaker than that of temperature. The contribution of other factors was insignificant; the first two axes of CCA accounted for nearly 79% of the variance.

**Table 2:** Presence (+) or absence (–) of periodicities in the abundances of dominant plankton taxa, total holo- and meroplankton biomass according to continuous wavelet transform analysis.

Species	Periodicity	2 year	3 year	4 year	6 year	8 year	Strong signal present, years
HOLOPLANKTON							
*Acartia clausi*	Twofold-year	+	–	+	–	+	Since 1998
*Aurelia aurita*, adults	Twofold-year	+	–	+	–	+	Until 2002
*Aurelia aurita*, larvae	Twofold-year	+	–	+	–	+	Until 2002
*Beroe ovata*, adults	Twofold-year	+	–	+	–	+	Since 2002
*Beroe ovata*, larvae	Twofold-year	+	–	+	–	+	Since 2002
*Calanus euxinus*	Twofold-year	+	–	+	–	+	Since 1998
*Centropages ponticus*	Twofold-year	+	–	+	–	+	Since 1998
Copepoda, nauplii	Blurred +	+	+	+	+	+	Since 1998
*Evadne nordmanni*	Twofold-year	+	–	+	–	+	Since 1998
*Mnemiopsis leidyi* <2 mm	Twofold-year	+	–	+	–	+	Until 1998
*Mnemiopsis leidyi* ≥2 mm	Twofold-year	+	–	+	–	+	Until 2002
*Noctiluca scintillans*	Twofold-year	+	–	+	–	+	Since 2002
*Oikopleura dioica*	Twofold-year	+	–	+	–	+	Since 1998
*Oithona davisae*	Twofold-year	+	–	+	–	+	Since 2010
*Oithona similis*	Twofold-year	+	–	+	–	+	Always
*Paracalanus parvus*	Twofold-year	+	–	+	–	+	Since 1998
*Parasagitta elegans*	Twofold-year	+	–	+	–	+	Since 1998
*Penilia avirostris*	Twofold-year	+	–	+	–	+	Since 1998
*Pleopis polyphemoides*	Twofold-year	+	–	+	–	+	Since 1998
Polychaeta, larvae	Twofold-year	+	–	+	–	+	Since 1998
*Pseudevadne tergestina*	Twofold-year	+	–	+	–	+	Since 1998
Rotifera	Blurred +	+	+	+	+	+	Since 1998
Surface chlorophyll a	Twofold-year	+	–	+	–	+	Since 1998
Total holoplankton biomass	Twofold-year	+	–	+	–	+	Since 1998
MEROPLANKTON							
Bivalvia, larvae	Fourfold-year	–	–	+	–	+	Since 1998
Gastropoda, larvae	Fourfold-year	–	–	+	–	+	Since 2002
Phoronidae, larvae	Fourfold-year	–	–	+	–	+	Since 1998
Cirripedia, cypris stage	Blurred	+	+	–	+	+	Since 1998
Cirripedia, nauplii	Blurred +	+	+	–	+	+	Since 1998
Decapoda, larvae	Blurred +	+	+	–	+	+	Since 2002
Harpacticoida	Blurred +	+	+	–	+	+	Since 1998
Pisces, larvae	Blurred +	+	+	+	+	+	Always
Pisces, ova	Blurred +	+	+	+	+	+	Always
Trachymedusae, larvae	Blurred +	+	+	+	+	+	Since 2000
Total meroplankton biomass	Blurred +	+	+	+	+	+	1998–2002

**Note:**

1-year periodicities are present in all taxa and not shown.

Most taxa including biomass of the holo- and meroplanktonic animals were grouped in the upper left part of the CCA plot, showing positive response to the increase in temperature and *Chl*. Negative response to both dominant factors was shown by *N. scintillans* and Rotifera.

**Figure 4:**
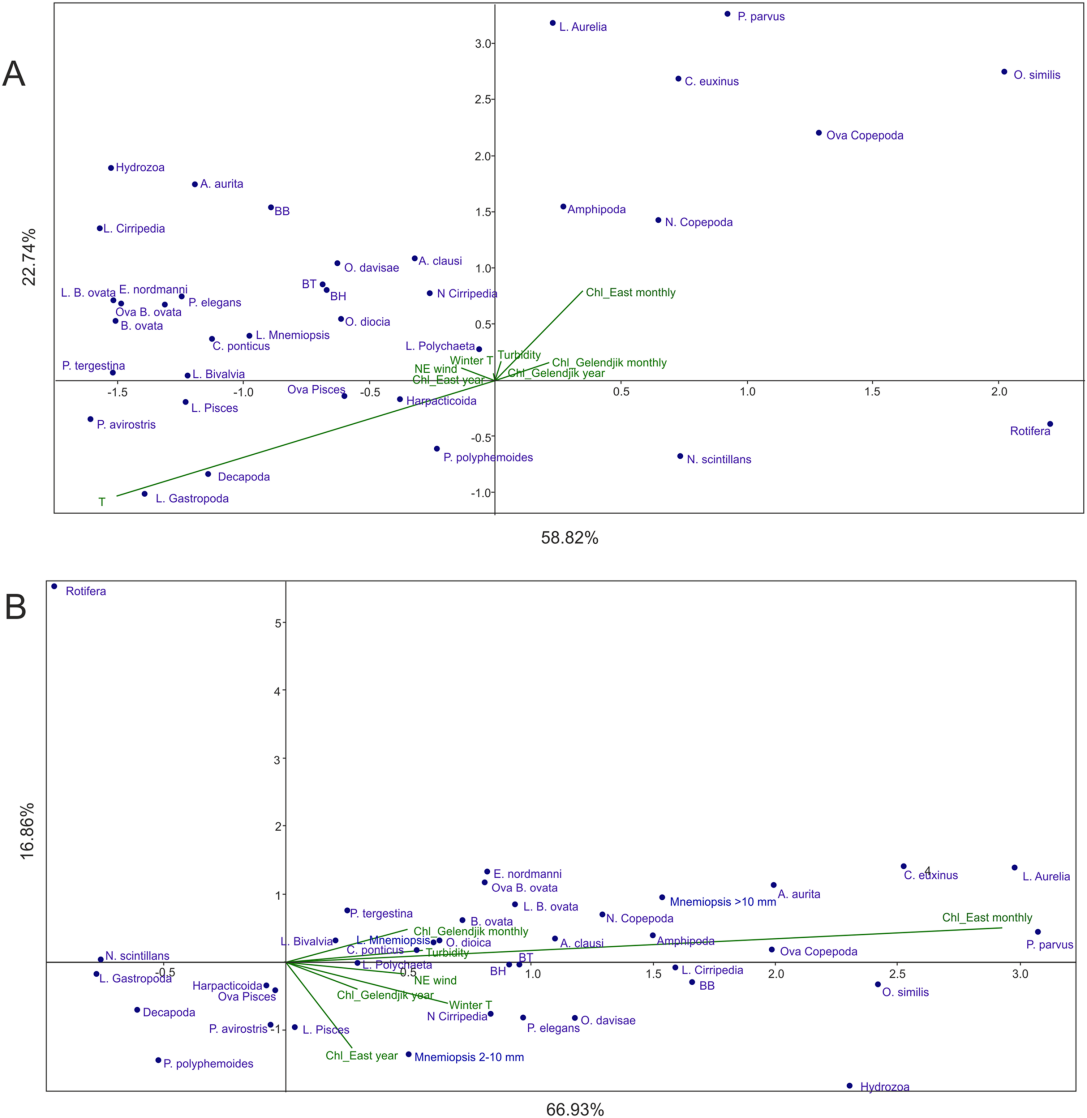
CCAs of samples taken in 1998–2017 with abundances of *M. leidyi* considered as dependent variables. (A) Samples with all environmental variables included, (B) samples with temperature (seasonal signal) excluded. Two first axes (F) with respective explained variance represented. Taxa code seen in Table 2; BH, BB, and BT: biomass of the holoplankton, meroplankton, and total plankton biomass, respectively. Environmental variables: temperature (T), *Chl* averaged over the Gelendjik area over 1 month (Chl Gelendjik monthly), (2) *Chl* averaged over the Gelendjik area over 1 year (Chl Gelendjik year), *Chl* averaged over the central East area over 1 month (Chl East monthly), *Chl* averaged over the central East area over 1 year (Chl East year), Northeast wind (NE wind), turbidity (Turbid), lowest winter temperature (Winter T).

Analysis 2 (*M. leidyi*-linked parameters transferred from environmental factors to dependent variables) provided results similar to Analysis 1 (Fig. 4A): Factor 1 was mainly explained by temperature, while Factor 2 by *Chl* averaged over 1 month and over the open East Black Sea, both factors explaining nearly 82% of the variance. As in Analysis 1, *Chl* averaged over the open East Black Sea provided a much stronger signal than *Chl* averaged over Golubaja Bay. The same most taxa-rich cluster of taxa, now with three *M. leidyi*, was positioned in the upper left part of the plot (positive response to the increase of temperature and of *Chl*) and same two taxa were in the lower right quarter (negative response).

Analysis 3 (the strongest signal, that is, temperature, removed) grouped the taxa as a function of productivity in the open East Black Sea (Fig. 4B). Indeed, Factor 1 was mostly linked to *Chl* averaged over 1 month, while Factor 2 was primarily associated with *Chl* averaged over 1 year (Fig. 4B). Contribution of other factors was much less significant; both factors accounted for nearly 77% of the variance.

Analysis 4 (the *Chl*-recording satellites did not act but *M. leidyi* signals included as an environmental factor in the period of the uncontrolled growth until 1998) showed results (Fig. 3B) significantly different from those of Analysis 1 (Fig. 3A). Two subequal environmental parameters were dominant, both explaining mainly Factor 1 (~67% of variance): temperature and abundance of *M. leidyi* larvae. The *M. leidyi* signal was only 1.5 times weaker than the seasonal signal. *Y*-axis explained an insignificant part of the variance (~17%) and was additionally associated with Northeast winds.

### Long-term periodicities: CWT analyses

**Figure 5:**
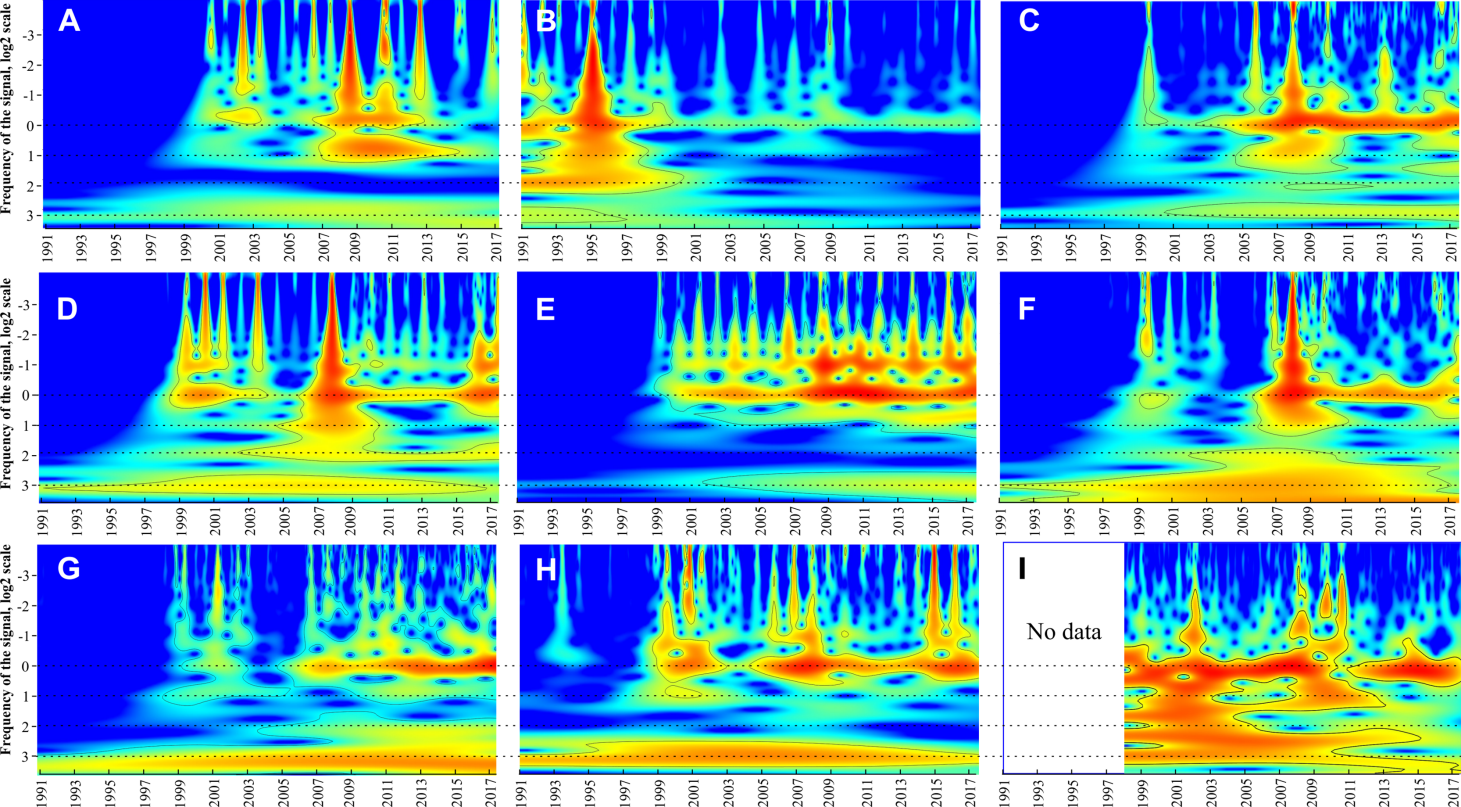
Continuous wavelet transform. *Beroe ovata* (A), *Mnemiopsis leidyi* (B), *Centropages ponticus* (C), *Pseudevadne tergestina* (D), *Pleopis polyphemoides* (E), *Oikopleura dioica* (F), *Acartia clausi* (G), total holoplankton biomass (H), surface chlorophyll concentrations (I). The vertical axes in the plots are in logarithmic size scales (base 2). Signal power is shown in colour. Horizontal dotted lines indicate visible periodicities.

Continuous wavelet transform showed that all taxa have a statistically significant (*p* < 0.05) band corresponding to 1-year periodicity (near zero on *y*-axis in Fig. 5) and one to three weaker bands between 10^−2^ and 10^−1^ year corresponding to within-year fluctuations. For all groups except *M. leidyi* 1-year band was absent before 1999–2000 and distinctive after 2000. Conversely, 1-year band of *M. leidyi* was strong before 1999–2000 and weak after this period. In addition to these, other bands occur and group the taxa into three clusters (Table 2):

1. The most abundant complex of the holoplankton having a 2^n^ periodicities (2, 4, 8 years). Some bands may be weak (e.g. a 2-year band in *Penilia avirostris* or 4-year band in *Pleopis polyphemoides*) but still distinct. Same periodicities were observed for the total holoplankton biomass and *Chl*.
2. The larvae of benthic taxa such as Bivalvia, Gastropoda, and Phoronidae having 4-year periodicities: 4 and 8 year.
3. Taxa, which were not identified to species level, showing nearly all possible bands: Harpacticoida, larvae of Cirripedia, Decapoda. No definite periodicity was observed for the total meroplankton biomass

Continuous wavelet transforms and Table 2 (last column) suggested three states of the Black Sea ecosystem in the period 1991–2017: 1991–1998, 1999–2002, and 2003–2017. In the first period, most taxa did not show significant signals of their long-term periodicity in CWTs (Fig. 5), while signals of *M. leidyi* were very strong; *B. ovata* was absent. During the second period, signals of most taxa including *B. ovata* appeared and increased, while the signals of *M. leidyi* significantly decreased. The third period, since 2003, is characterised by regular and strong signals in CWTs diagrams for most plankton taxa.

### Long-term periodicities: PERMANOVA and ANOSIM analyses

**Figure 6:**
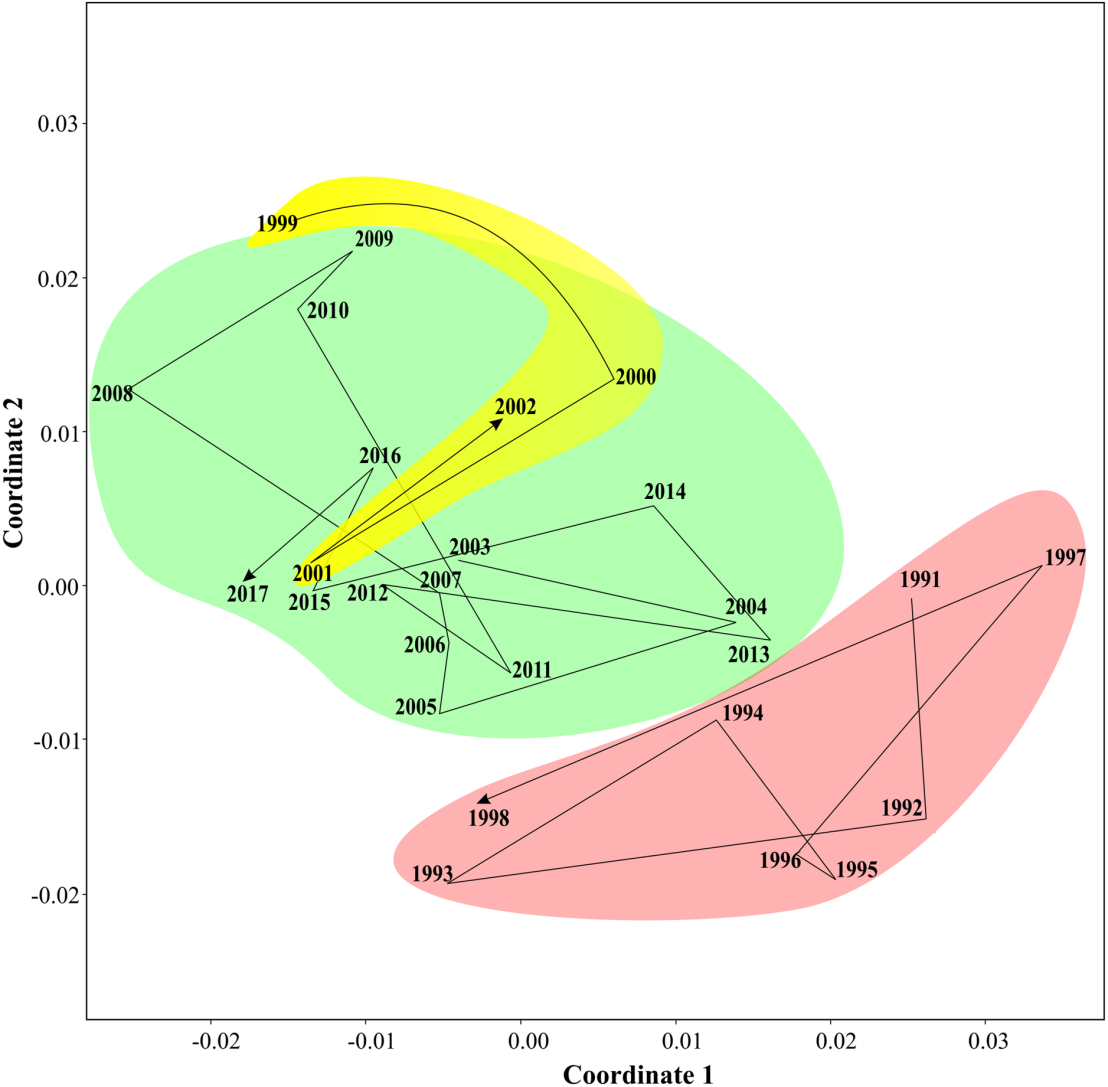
Results of MDS scaling (Bray–Curtis similarity index). The arrows show transitions between subsequent years, three states of coastal communities are in different colours.

Non-parametric tests supported three periods of the Black sea coastal ecosystem. One-way ANOSIM, with Kulzhinsy similarity index and one-way PERMANOVA with Bray–Curtis index of similarity, revealed statistically significant differences between all three periods (*p* < 0.0001 in all cases). MDS diagram (Fig. 6) showed three community states corresponding to three periods. The arrows show transitions between communities in subsequent years. The first period was very distinct from the two following ones, while the second and the third periods were closer to each other.

### Multiple and bivariate regressions

We calculated multiple and bivariate linear regressions (Table 3) between abundances of plankton taxa and the biomass values from one side (dependent variables) and two major environmental factors that explained the main bulk of the variance according to CCAs on the other: surface temperature and surface chlorophyll averaged over the open East Black Sea and over 1 month.

**Table 3:** Statistics of multiple linear regressions between abundances (ind m^−3^) and biomass (mg m^−3^) of plankton taxa as dependent variables and two independent variables: surface chlorophyll averaged over the East Black Sea and over 1 month.

Taxa	Multiple *R*	ANOVA, *F*	*p*	Coefficient *T*	Coefficient *Chl*	Constant
HOLOPLANKTON						
*Acartia clausi*	0.209	15.676	2.201E-7	10.924	109.250	−136.200
*Aurelia aurita*, adults	0.116	4.691	0.009	0.010	0.176	−0.242
*Aurelia aurita*, larvae	0.085	2.496	0.083	n/a	n/a	n/a
*Beroe ovata*, adults	0.199	14.159	9.422E-7	0.016	0.048	−0.219
*Beroe ovata*, larvae	0.185	12.134	6.624E-6	0.542	6.256	−10.736
*Beroe ovata*, ova	0.156	8.590	2.066E-4	0.071	0.974	−1.501
*Calanus euxinus*	0.161	9.116	1.237E-4	−0.005	2.050	−0.666
*Centropages ponticus*	0.253	23.508	1.331E-10	6.070	31.027	−86.226
Copepoda, nauplii	0.040	0.551	0.577	n/a	n/a	n/a
Copepoda, ova	0.047	0.747	0.474	n/a	n/a	n/a
*Evadne nordmanni*	0.109	4.138	0.016	1.759	10.588	−28.898
Hydrozoa	0.180	11.535	1.181E-5	0.950	12.495	−20.018
*Mnemiopsis leidyi* <2 mm	0.275	28.085	1.885E-12	0.405	−0.594	−3.399
*Mnemiopsis leidyi* >10 mm	0.183	11.885	8.422E-6	0.063	0.713	−1.282
*Mnemiopsis leidyi* 2–10 mm	0.148	7.665	0.001	0.147	−0.898	−1.287
*Noctiluca scintillans*	0.114	4.524	0.011	−12.502	−1517.5	1964
*Oikopleura dioica*	0.179	11.356	1.406E-5	3.228	22.781	−41.122
*Oithona davisae*	0.161	9.164	1.181E-4	0.515	0.825	−4.851
*Oithona similis*	0.178	11.237	1.576E-5	−1.729	4.715	37.658
*Paracalanus parvus*	0.211	16.139	1.413E-7	−1.232	130.700	−4.870
*Parasagitta elegans*	0.303	34.719	4.316E-15	2.492	20.907	−41.857
*Penilia avirostris*	0.377	57.139	1.103E-23	40.701	−4.780	−472.01
*Pleopis polyphemoides*	0.180	11.483	1.243E-5	5.613	−144.84	91.634
*Pseudevadne tergestina*	0.180	11.515	1.205E-5	4.384	25.598	−69.500
Rotifera, larvae	0.127	5.637	0.004	−5.213	−108.450	198.62
Holoplankton biomass	0.319	38.929	1.628E-17	1.297	7.500	−16.146
MEROPLANKTON						
Amphipoda	0.035	0.425	0.654	n/a	n/a	n/a
Bivalvia, larvae	0.308	36.177	1.151E-15	12.92	22.73	−155.96
Cirripedia, cypris stage	0.253	23.437	1.423E-10	0.727	8.133	−14.446
Cirripedia, nauplii	0.152	8.107	3.312E-4	4.151	11.491	−26.701
Decapoda, larvae	0.295	32.757	2.577E-14	0.370	−2.948	−1.696
Gastropoda, larvae	0.207	15.414	2.828E-7	6.217	−28.164	−49.326
Harpacticoida	0.236	20.368	2.548E-9	0.461	−6.927	2.795
Polychaeta, larvae	0.149	7.809	4.43E-4	1.554	−13.719	11.802
Pisces, larvae	0.308	36.063	1.276E-15	0.019	−0.027	−0.184
Pisces, ova	0.234	19.902	3.967E-9	0.061	−0.539	−0.058
Meroplankton biomass	0.105	3.813	0.023	0.051	0.834	−1.082
Total plankton biomass	0.327	41.174	1.289E-17	1.347	8.333	−17.228

**Note:**

For all taxa *N* = 691, ANOVA, d*f*1, d*f*2 = 2.688. Bivariate regressions between taxa and each of independent variables were tested; shaded areas indicate variables not passed *t*-test.

Most taxa passed the ANOVA test, and multiple regressions were statistically significant with the exception of the following taxa: larvae of the medusa *A. aurita*, unidentified copepod ova and nauplii, and amphipods.

Some taxa passed the ANOVA test for multiple regressions but did not pass the *t*-test for bivariate regressions. Some of them were robustly correlated with temperature but not with *Chl*: the ctenophores *B. ovata* (adults) and *M. leidyi* (larvae and juveniles), fine filter feeders *Oikopleura dioica*, *Centopages ponticus*, *Penilia avirostris*, *Evadne nordmanni*, both species of *Oithona*, fish ova and larvae, larvae of several benthic taxa (bivalves, gastropods, polychaetes, cirripeds) and the total meroplankton biomass. Conversely, three taxa were robustly correlated with *Chl* but not with temperature: the protozoan *N. scintillans*, and the copepods *Paracalanus parvus* and *Calanus euxinus*.

## Discussion

### Herarchy of driving environmentals: recent state

Recent (since 1998) structure of the coastal zooplankton communities is mostly explained by two factors: temperature (seasonal signal) and surface *Chl* (productivity signal) (Analysis 1, Fig. 3A). The signal of *M. leidyi* is minor and not comparable with both major factors. Moreover, the vectors linked to *M. leidyi* (including the strongest signal of larvae) are nearly coaxial to the temperature vector, which may suggest their strong dependence on temperature. A transfer of *M. leidyi* abundances from the environmentals to a set of dependent variables (Analyses 3–4) is therefore reasonable for the period since 1998.

Analysis 2 provides more robust evidence for the two main factors driving plankton composition and explaining over 80% of variance. The strongest factor is temperature driving succession of plankton communities. The second factor, as in Analysis 1, is a productivity signal. We used average surface *Chl* concentrations (satellite data) as a proxy for surface productivity and tested four combinations of averaging: over the local coastal area (0.25° 0.25°, both over 1-month and over 1-year periods), and over the open part of the East Black Sea (1° × 1°, both over 1-month and over 1-year periods). Averaging over the local area provided weaker signals on both time scales (Fig. 4A).

Strongest *Chl* signals from a large open area may highlight a typical spatial scale of the processes providing energy transfer from primary producers to the zooplankton in the East Black Sea. The dominant spatial scales for phytoplankton growth in the temperate Atlantic were estimated as 40–120 km (Flagg, Wirick & Smith, 1994), that is similar to the 1° × 1° area used in our studies. Smaller spatial scales, especially in the coastal areas affected by the riverine runoff, may mask the productivity signal. It is noteworthy that similar, and even rougher, spatial averaging (5° × 5°) provided robust correlations between surface *Chl* and basic zooplankton groups in the open Atlantic waters (Vereshchaka et al., 2016, 2017; Vereshchaka, Lunina & Sutton, 2019).

Our data also show the importance of the averaging period: 1-month periods provided much stronger signals regardless the spatial averaging scales (Fig. 4A), which suggests comparatively fast (within 1 month) mesoplankton response to the productivity increase in temperate waters. Interestingly, in the tropical and equatorial waters of the Atlantic, the averaging of the surface *Chl* over 1 year provided stronger signals than in the temperate Black Sea (Vereshchaka et al., 2016, 2017; Vereshchaka, Lunina & Sutton, 2019). The (sub)tropical communities do not experience rapid seasonal changes, and a larger temporal scale is more appropriate to assess their structure.

Analysis 3, with further removal of the strongest signal (temperature), supports the results of Analyses 1 and 2 concerning an almost negligible contribution of the rest of the environmental factors except surface productivity. As in previous analyses, *Chl* averaging over 1 month and over a 1° × 1° area provided the strongest signal. However, the year-averaged *Chl* was also visible (associated with the second axis—Fig. 4B), thus showing some importance of the general productivity background on a wider temporal scale (as averaged over a year).

Surprisingly, the northeast wind did not significantly contribute to the changes in the plankton composition. This wind, which is directed seawards, usually results in a negative surge and a decrease in surface temperature of 1–2 °C. Increased turbidity also did not provide a significant signal. Thus, both local factors such as the wind and the turbidity did not explain any significant variations in the structure of the plankton communities, which suggests that the coastal Black Sea plankton is not strongly affected by local events. The plankton in the sampling area likely represents a rather homogenous coastal community that is primarily affected by seasonal and basin productivity factors.

Minimum winter temperature, which was shown to be a significant driver of the spring phytoplankton bloom (Mikaelyan et al., 2017), was not significant as well (Fig. 4B). Spring blooms may affect plankton composition for a short period, while their impact is weak on a larger timescale.

### Herarchy of driving environmentals: the period of the uncontrolled invasion of *M. leidyi*

The hierarchy of the environmental factors was, however, different not long ago. One of the most drastic events in the recent history of the Black Sea was an invasion of the carnivorous ctenophore *M. leidyi* (late 1980s), which caused a degradation of the pelagic ecosystem. Abundances of *M. leidyi* started to decline and pelagic ecosystems began to restore in the East Black Sea only in 1999, after the invasion and mass development of the ctenophore *B. ovata*, a predator of *M. leidyi* (Finenko et al., 2003; Shiganova et al., 2004; Arashkevich et al., 2008; Vinogradov et al., 2005). These events are visible in our dataset at a temporal boundary of 1998: comparison of the data before 1998 and after this period (Figs. 3A and 3B) shows a significant weakening of the *M. leidyi*-linked factors as compared to the dominant temperature signal. Before the invasion of *B. ovata*, which now controls the population of *M. leidyi*, the *M. leidyi*–linked signal was almost as strong as the temperature signal. Unlike the recent state, when the *M. leidyi* signal is relatively weak (Analysis 1, Fig. 3A), in the period of the uncontrolled bloom *M. leidyi* explained a significant part of the variance.

Among the three *M. leidyi*-associated variables (abundances of larvae, juveniles and adults), larvae produced the strongest signal both before and after 1998. A small plankton net which we used may not sample larger size groups representatively that may mask their proper signal.

Our data thus show that in the periods of uncontrolled invasions immigrants may become a strong environmental factor comparable to seasonal signals such as temperature. When the invasion is taken under control (in the Black Sea, the population of *M. leidyi* by that of *B. ovata* since 1999), the invader explains much smaller part of the variance.

One additional phenomenon in the recent past concerns a wind signal, which was conspicuous in the period of *M. leidyi* bloom and mostly explained Factor 2 (Fig. 3B). In situ studies have shown that the vertical migrations of gelatinous plankton in the coastal waters of the Black Sea are synchronised with winds and may favour aggregations of the ctenophores near the shores (Vereshchaka, 2002a, 2002b). These wind-driven aggregations may not be a significant factor since 1998, but could be much more important in the period of the uncontrolled bloom of *M. leidyi*.

### Long-term periodicities

Spectral analyses show 1-year periodicities in all plankton taxa, they are apparently related to the seasonal forcing and thus trivial. More interesting is the presence of other statistically significant periodicities: twofold-year periodicities for most holoplanktonic taxa (Table 2). Same periodicities were also recorded and statistically robust for the whole plankton biomass and *Chl*. It is the biannual *Chl* fluctuations which likely force and explain interannual variations of dominant plankton taxa and the total plankton biomass. Our *Chl* data derive from the central open waters in the eastern Black Sea, which are laying maximally aside of the coast and where the most intensive bottom-up flow of nutrients to the photic zone defines the ecosystem functioning in the most ‘pure’ form (Oğuz, 2008). Mechanisms of the interannual chlorophyll variations are far from being understood. First attempts to explain amplitude of the spring phytoplankton blooms have been done recently (Mikaelyan et al., 2017) and showed its relation to winter convection. Other drivers of the year-to-year fluctuations of productivity remain unclear and possible mechanisms of the observed biannual *Chl* fluctuations merit future analyses.

Another periodicity (4 years—Table 2) is characteristic for some meroplanktonic larvae: Bivalvia, Gastropoda, Phoronidae. This type of periodicity may reflect population waves in the benthic assemblages of the adults. The last type of periodicities (nearly all possible bands in CWT) refers to speciose taxa, which were not identified to species (Table 2). Individual species within these taxa may have distinct periodicities, which, however, interfere and mask each other in our dataset.

It is noteworthy that the 8-year long-term cycle that is evident in most groups, but especially in *Oikopleura dioica*, *Acartia clausi*, total holoplankton, and meroplankton, may be a consequence of the invasion of *M. leidy*.

### Three states of the coastal communities in 1991–2017: a possible presence of a transitional period?

Based on changes of the total phytoplankton biomass, three periods of the Black Sea ecosystem have been reported previously: pre-eutrophication in 1969–1983, eutrophication in 1984–1995, and post-eutrophication in 1996–2008 (Mikaelyan, Zatsepin & Chasovnikov, 2013). Our data encompass only the two last periods and do not show any change in 1995–1996 that suggests insignificant impact of eutrophication on the structure of plankton communities. Instead, we have revealed two boundaries, 1998–1999 and 2002–2003, first of which well correspond to the time of appearance of *B. ovata* in the Eastern Black Sea (1999) and following recovery of the plankton communities after the shock linked to the invasion of *M. leidyi*.

Indeed, before the *M. leidyi* invasion, the zooplankton biomass in the Blue Bay averaged over a year reached 48 mg m^−3^ (wet weight: Pasternak, 1983) and showed two seasonal peaks. The first peak was linked to the spring phytoplankton bloom and caused by the reproduction and development of dominant eurythermal copepods such as *Pseudocalanus elongatus, Calanus euxinus, Oithona similis*, and *Paracalanus parvus*. The second peak occurred in August–September and was mainly associated with cladocerans, the copepod *Centopages ponticus*, and meroplanktonic larvae of benthic species (Pasternak, 1983). After *M. leidyi* invasion, the zooplankton biomass decreased by more than one order of magnitude:1.2–2.3 mg m^−3^ wet weight (Khoroshilov & Lukasheva, 1999) and the zooplankton composition has changed (Vinogradov et al., 1991; Gruzov, Lyumkis & Napedovskiy, 1994). The most visible changes were linked to a great decrease in abundances of many taxa, especially cladocerans and chaetognaths (Vinogradov, Shiganova & Khoroshilov, 1995; Zaitsev, 1992). This period was recorded on our CWTs diagrams as a period of strong annual signals of *M. leidyi* population and lack of signals of most other plankton taxa (a uniform colour fill—Fig. 5). That means that in this period, that is the period 1991–1998 in our set, most taxa and the total zooplankton biomass were greatly suppressed and had no significant peaks of abundance.

Conversely, the recent period since 2002 is characterised by the strong 1-year signals of most taxa and of the total biomass and even presence of the two peaks (periodicity ½ year in Fig. 5). This may indicate a new, balanced state of the ecosystem with two annual peaks of the total biomass, as it was before the invasion of *M. leidyi*. The recent period have been described on the sub-basin scale studies both in the West (Stefanova et al., 2005) and in the North–East Black Sea (Arashkevich et al., 2008; Vinogradov et al., 2005) and revealed a recovery of zooplankton communities as compared with the early 1990s. At present, the populations of the cladocerans and chaetognaths have restored and significantly contribute to the zooplankton community again (Moncheva et al., 2012), similar to that observed in 1970–1980s (Greze, 1979; Sorokin, 1982).

In addition to these two outlied periods, our data show a new one, which was not recorded before. There was a transitional period 1999–2002 characterised by weakening signals linked to the *M. leidyi* peaks and increasing signals of other dominant taxa including *B. ovata* and the total zooplankton biomass.

All three periods, including the transitional period 1999–2002, are visible on CWTs diagrams and gain statistical support. These periods are likely associated with the dominance of the ctenophore *M. leidyi* until 1999 (first period), and with the invasion and naturalising of the ctenophore *B. ovata*, which is controlling populations of *M. leidyi* since 1999. The transit between ecosystem states lasted nearly 4 years (1999–2002) and encompassed the years when the structure of coastal communities was changing from the state 1991–1997 to the state 2003–2017 (Fig. 5). It is noteworthy that the transitional period was marked by the strongest signal of *A. aurita* indicating highest abundances and maximal peaks of this species, which might take advantage in this period. We thus can assess a period, which is required for a pelagic ecosystem recovery after invasions: 4 years in a case of *B. ovata*.

### Regressions

The two strongest factors, temperature and surface *Chl*, explain ~80% of the variance, and each can be used for regression analyses alone. However, multiple regressions (with both factors included) provide more robust correlations than do bivariate regressions (with a single factor included). Indeed, such taxa as *N. scintillans, Paracalanus parvus*, and *Calanus euxinus* do not show significant correlation with temperature, while addition of *Chl* makes the correlations robust. Conversely, an even wider range of taxa, including part of ctenophores (adults of *B. ovata*, larvae and juveniles of *M. leidyi*), fine feeders (*Centopages ponticus, Oikopleura dioica, Penilia avirostris, E. nordmanni*), both species of *Oithona*, fish ova and larvae, are not correlated with *Chl*, while addition of the second parameter, temperature, makes correlations statistically significant. We failed to find explanations for why any given taxa are better correlated with either temperature or *Chl*. For example, fine feeders may be correlated with *Chl* and not with temperature (*Paracalanus parvus*), or vice versa (*Centopages ponticus, Oikopleura dioica, Penilia avirostris, E. nordmanni*). Moreover, different size groups of the same species may be well correlated with both environmentals (adults of *M. leidyi*, ova and larvae of *B. ovata*) or with only a single one (adults of *B. ovata*, ova and larvae of *M. leidyi*).

It is noteworthy that many meroplankton taxa, as well as the total meroplankton biomass, are not correlated with *Chl:* larvae of the medusa *A. aurita*, larvae of Bivalvia, Gastropoda, Polychaeta and Cirripedia (nauplii). Life cycles of these groups may rather be controlled by seasonal benthic processes than by the surface productivity (*Chl*). An addition of the second factor (temperature) makes multivariate correlation robust for all these taxa except larvae of *A. aurita*.

Both uni- and bivariate analyses do not provide robust correlations for copepod ova and nauplii and for amphipods. These taxa were represented by several species, which had different life cycles and thus superimposed and masked possible individual regressions.

Overall, the regression analyses show that abundances and biomass of most taxa, including the total holo- and meroplankton biomass, may be satisfactorily explained by two variables: temperature and *Chl*. Correlations are generally less robust for the meroplankton, but are highly satisfactory for many holoplanktonic taxa and, what is more important, for the total plankton biomass (*p* = 1.289 × 10^–17^). Based on such robust correlations, coefficients obtained for the bivariate regressions (linear model) enable the forecast of plankton abundances and biomass on the basis of current temperature and *Chl* data. Although generally low but still statistically significant for many taxa, *R* is remarkably high for the total plankton biomass. Thus, the total amount of wet organic within mesoplankton coastal communities is robustly and predictably linked to temperature and productivity, while abundance and biomass values of individual taxa are more variable.

## Conclusions

Currently, temperature and productivity are the major environmental factors driving zooplankton dynamics. The best productivity signal is obtained if the *Chl* data are averaged over an open sea and over 1 month. Not long ago, before 1999, abundance of *M. leidyi* was one of the major factors explaining the zooplankton variance. Spectral analysis of species abundances revealed a 4-years transitional period in 1999–2002 (not reported before) when ecosystem adapted to a new invader, *B. ovata*. Statistically robust 2- and 3-year periodicities were retrieved for most plankton taxa and some benthic larvae. We found robust correlations between temperature and surface chlorophyll concentration on one side and plankton abundances and biomass on the other, and retrieved multivariate regressions, which may have a prognostic value total plankton biomass. The total mesoplankton biomass is robustly and predictably linked to temperature and productivity, while abundance and biomass values of individual taxa are more variable.

## Supplemental Information

10.7717/peerj.7588/supp-1 Supplemental Information 1 Data matrix of Black Sea coastal zooplankton, 1991–2016.

## Additional Information and Declarations

### Data Availability

The following information was supplied regarding data availability: The raw data is available as a Supplemental File.
